# Anemia and Blood Biomarkers of Alzheimer Disease in Dementia Development

**DOI:** 10.1001/jamanetworkopen.2026.4029

**Published:** 2026-04-17

**Authors:** Martina Valletta, Davide Liborio Vetrano, Chengxuan Qiu, Marco Canevelli, Edoardo Miccoli, Sarah Andersson, Claudia Fredolini, Giuseppe Bruno, Bengt Winblad, Laura Fratiglioni, Giulia Grande

**Affiliations:** 1 Aging Research Center, Department of Neurobiology, Care Sciences and Society, Karolinska Institutet and Stockholm University, Stockholm, Sweden; 2 Stockholm Gerontology Research Center, Stockholm, Sweden; 3 Department of Human Neuroscience, Sapienza University, Rome, Italy; 4 Affinity Proteomics Stockholm, Science for Life Laboratory, Department of Protein Science, School of Engineering Sciences in Chemistry, Biotechnology and Health (CBH), Royal Institute of Technology (KTH), Solna, Sweden; 5 Division of Neurogeriatrics, Department of Neurobiology, Care Sciences and Society, Karolinska Institutet, Solna, Sweden; 6 Theme Inflammation and Aging, Karolinska University Hospital, Huddinge, Sweden

## Abstract

**Importance:**

Anemia has been associated with increased dementia risk, but its relationship with Alzheimer disease (AD) blood biomarkers remains unclear.

**Objective:**

To investigate whether there is a cross-sectional association between hemoglobin and AD blood biomarker levels and a longitudinal association of hemoglobin and AD biomarkers with incident dementia.

**Design, Setting, and Participants:**

This cohort study used data from the Swedish National Study on Aging and Care in Kungsholmen (SNAC-K), a longitudinal population-based study. Randomly selected adults aged 60 years or older were enrolled at baseline (March 21, 2001, to August 30, 2004) and followed up every 3 or 6 years according to age through December 31, 2019. Data analysis was conducted between September 1, 2024, and January 7, 2026.

**Exposures:**

Hemoglobin level measured at baseline. Anemia was defined according to World Health Organization criteria.

**Main Outcomes and Measures:**

Incident dementia, diagnosed according to *Diagnostic and Statistical Manual of Mental Disorders, Fourth Edition* (*DSM-IV*) criteria, and serum concentrations of phosphorylated tau 217 (p-tau217), neurofilament light chain (NfL), and glial fibrillary acidic protein (GFAP), measured using Simoa assays. Cox proportional hazards regression and quantile regression models were used to examine the association of hemoglobin with dementia risk and AD blood biomarkers. The joint association of hemoglobin and AD blood biomarkers with dementia development using Cox proportional hazards regression was also explored.

**Results:**

A total of 3363 adults were enrolled in SNAC-K (73.3% participation rate). After excluding those with dementia or missing AD biomarkers or hemoglobin measures, 2282 dementia-free participants were included in the current study (median age, 72.2 [IQR, 60.8-81.1] years; 1406 [61.6%] female). During a mean (SD) follow-up of 9.3 (4.3) years, 362 participants (15.9%) developed dementia. Compared with individuals with a normal hemoglobin level, those with anemia had higher baseline levels of p-tau217 (β, 0.22; 95% CI, 0.15-0.30), NfL (β, 0.25; 95% CI, 0.19-0.31), and GFAP (β, 0.08; 95% CI, 0.03-0.12) and showed a higher risk of developing dementia (hazard ratio [HR], 1.66; 95% CI, 1.21-2.28) during follow-up. Participants with both anemia and high p-tau217, NfL, or GFAP had the highest hazard of dementia (eg, adjusted HR of 3.64 [95% CI, 2.39-5.56] among those with anemia and high NfL).

**Conclusions and Relevance:**

In this cohort study of dementia-free older adults, anemia was associated cross-sectionally with higher levels of AD blood biomarkers and longitudinally with increased dementia risk. The highest dementia risk occurred when low hemoglobin and elevated AD biomarkers coexisted, suggesting a potential interplay between anemia and neuropathology in dementia development.

## Key Points

**Question:**

Is late-life anemia associated with blood biomarkers reflecting Alzheimer disease pathology, neurodegeneration, and glial activation and with dementia risk?

**Findings:**

In this cohort study of 2282 dementia-free adults aged 60 years or older, anemia was cross-sectionally associated with higher levels of phosphorylated tau 217, neurofilament light chain, and glial fibrillary acidic protein and longitudinally associated with a significantly higher risk of incident dementia over a mean (SD) follow-up of 9.3 (4.3) years. The highest dementia risk occurred when anemia and elevated biomarkers coexisted.

**Meaning:**

These findings suggest that anemia may interact with neuropathologic processes, potentially accelerating dementia development.

## Teaser

This cohort study assesses whether anemia among adults aged 60 years or older is associated with incident dementia risk and blood biomarkers of Alzheimer disease pathology, neurodegeneration, and glial activation.

## Introduction

Anemia is a common condition in old age, affecting approximately 10% of individuals aged 65 years or above in the US,^1^ with prevalence increasing with age.^2^ Anemia has been associated with several adverse health outcomes,^1,2^ including increased dementia risk.^3,4,5^ Several mechanisms have been proposed to explain the association between anemia and cognitive deterioration. One hypothesis suggests that anemia leads to chronic cerebral hypoxia and oxidative stress, which may contribute to neuronal loss, vascular dysfunction, and astrocytic activation.^6^ Indeed, neuroimaging studies have found brain atrophy^7^ and accumulation of white matter hyperintensities in individuals with anemia.^3,7^ Although evidence remains limited, an association between anemia and Alzheimer disease (AD) pathology has also been reported.^8^

Blood biomarkers of AD have recently emerged as reliable proxies of AD pathology, as they highly correlate with in vivo measures such as cerebrospinal fluid (CSF) and brain positron emission tomography (PET) markers.^9,10^ In addition, blood levels of phosphorylated tau 217 (p-tau217), neurofilament light chain (NfL), and glial fibrillary acidic protein (GFAP) reflect early pathologic changes in cognitively unimpaired individuals long before dementia diagnosis.^11^ These findings support their use as a valid window into the brain to study neuropathologic changes. In a previous cross-sectional study, individuals with anemia had elevated levels of several AD blood biomarkers.^12^ However, whether these alterations reflect greater underlying neuropathology linked to the presence of anemia remains unclear. Investigating the interplay between anemia and AD blood biomarkers may enhance our understanding of how anemia relates to dementia development.

Using a well-characterized, population-based study comprising clinical and biological data from over 2000 older adults, we examined the association between hemoglobin levels, dementia risk, and AD blood biomarkers. We also explored the interplay between hemoglobin levels and neuropathology, as measured with AD blood biomarkers, in dementia development.

## Methods

### Study Population and Data Collection

This cohort study used data from the Swedish National Study on Aging and Care in Kungsholmen (SNAC-K),^13^ an ongoing longitudinal population-based study. At baseline (March 21, 2001, to August 30, 2004), SNAC-K enrolled individuals aged 60 years or older living in the Kungsholmen district of Stockholm. Participants were randomly selected across 11 age cohorts (60, 66, 72, 78, 81, 84, 87, 90, 93, 96, and ≥99 years) and followed up at intervals of 6 years (for those aged <78 years) or 3 years (for those aged ≥78 years) through December 31, 2019. At each study wave, clinical, laboratory, functional, and cognitive data were collected through medical examination, nurse interview, and cognitive assessment conducted by trained staff. All phases of SNAC-K were approved by the ethical committee at Karolinska Institutet and the regional ethical review board in Stockholm. All participants provided written informed consent to participate in the study and for use of their data for research purposes. No approval or additional consent was required for this study as it was based on analyses conducted within the scope of the original participants’ consent. The study’s results are reported following the Strengthening the Reporting of Observational Studies in Epidemiology (STROBE) reporting guideline for cohort studies.^14^

### Blood Biomarkers of AD

Peripheral venous blood samples were collected at baseline, and serum aliquots were stored at the Karolinska Institutet Biobank at −80 °C in cryogenic storage vials until analysis. Protein quantification was conducted at the Affinity Proteomics Stockholm Unit (SciLifeLab). Serum NfL and GFAP were measured using the Simoa Neurology 2-plex B assay kit (Quanterix). Serum p-tau217 was measured using the Simoa ALZpath p-Tau-217 Advantage PLUS assay kit (Quanterix). Measurements of p-tau217 below the limit of detection were replaced with a value of 0. Blood biomarkers were *z* scored based on baseline mean and SD to facilitate comparison between coefficients.

### Hemoglobin Measurement and Anemia Definition

Hemoglobin and mean corpuscular volume (MCV) were measured on blood samples collected at baseline at the St Göran’s Hospital laboratory. Anemia was defined following the World Health Organization criteria^15^ as blood hemoglobin level of 12 g/dL or less for females and 13 g/dL or less for males (to convert to g/L, multiply by 10.0). Based on MCV, anemia was classified as normocytic (MCV 80-100 fL), microcytic (MCV <80 fL), or macrocytic (MCV >100 fL). Hemoglobin levels were modeled using restricted cubic splines with 3 knots at the 10th, 50th, and 90th percentiles.

### Diagnosis of Dementia and Assessment of Mild Cognitive Impairment

Dementia was diagnosed at each study wave according to the *Diagnostic and Statistical Manual of Mental Disorders, Fourth Edition* (*DSM-IV*) criteria,^16^ following 3 sequential steps. The examining physician made a first preliminary diagnosis. A second preliminary diagnosis was made by a reviewing physician. In case of discordance, a final diagnosis was made by senior neurologists (L.F., G.G.) not involved in data collection. If a participant died between 2 SNAC-K visits, information was complemented through clinical medical records and the Swedish National Cause of Death Register to reduce the risk of death masking dementia. Mild cognitive impairment (MCI) was defined as scoring 1.5 SDs or more below the age-specific mean in at least 1 cognitive domain on a standardized neuropsychological battery, with preserved basic daily functioning, minimal impairment in instrumental activities, and absence of dementia.^17^

### Covariates

Completed educational level was categorized into elementary school, high school, or university or above. Chronic diseases were diagnosed through self-reports, physical evaluation, medical records, laboratory tests, and medication use and were coded following the *International Statistical Classification of Diseases and Related Health Problems, 10th revision* (*ICD-10*).^18^ Body mass index (BMI, calculated as weight in kilograms divided by height in meters squared) was measured at baseline; underweight was defined as BMI less than 18.5. Information on intake of iron and vitamin supplements (ie, vitamin B_9_, vitamin B_12_, and multivitamin combinations) was also collected. Blood interleukin 6 (IL-6) level was used as a measure of systemic inflammation. *APOE* genotyping was conducted, and participants were classified as *APOE* ε4 carriers if they had at least 1 *APOE* ε4 allele.

### Statistical Analysis

Data analysis was conducted between September 1, 2024, and January 7, 2026. Cox proportional hazards regression models were used to estimate associations of baseline hemoglobin level and anemia with incident dementia, using follow-up time as the time scale. Follow-up time was estimated from baseline until the occurrence of dementia, study withdrawal, or death, with deaths treated as censored observations. We tested the proportional hazards assumption by regressing the scaled Schoenfeld residuals against survival time, and we did not find any deviation. Quantile regression on the median was used to examine cross-sectional associations of hemoglobin level and anemia with AD blood biomarkers at baseline.

Cox proportional hazards regression models were then used to estimate the hazard of dementia associated with hemoglobin level, anemia, and AD blood biomarkers. AD blood biomarkers were modeled using restricted cubic splines with 3 knots (10th, 50th, and 90th percentiles) and also dichotomized using cutoffs previously derived in the same cohort.^11^

Analyses were adjusted for conditions known to be associated with anemia and dementia onset in older age and with AD blood biomarker levels.^12,19^ Basic adjustment included age cohort, sex, and educational level; additional adjustment factors included chronic kidney disease, heart diseases, cerebrovascular disease, cancer, underweight, intake of iron and vitamin supplements, and IL-6 level.

Given known sex differences in hemoglobin levels and anemia etiology,^1^ sex-stratified analyses were conducted, and interactions with sex were tested. We also tested interactions with *APOE* ε4 status and conducted stratified analyses. To restrict the analyses to cognitively unimpaired individuals at baseline and to minimize reverse causation, we conducted sensitivity analyses excluding participants with baseline MCI or those who developed dementia within the first 6 years of follow-up.

A 2-tailed *P* value less than .05 was considered statistically significant in all analyses. The statistical analyses were performed using Stata, version 17 (StataCorp LLC), and R, version 4.2.0 (R Project for Statistical Computing).

## Results

### Characteristics of the Study Population

From 3363 total SNAC-K participants (73.3% participation rate), we excluded 240 with dementia at baseline, 833 missing AD biomarkers, and 8 missing hemoglobin measures, leaving an analytic sample of 2282 participants (eFigure 1 in Supplement 1). Participants with missing data were older, more often female, and had a higher burden of chronic diseases than those included (eTable 1 in Supplement 1). The 2282 included participants had a median age of 72.2 years (IQR, 60.8-81.1 years); 1406 (61.6%) were females, 876 (38.4%) were males, and 826 (36.2%) had university education (Table 1). Participants were predominantly White, reflecting the demographic composition of the Kungsholmen district at the time of recruitment; detailed data on race or ethnicity in the cohort were not collected. At baseline, 199 participants (8.7%) had anemia, which in 180 cases (90.5%) was normocytic (hemoglobin levels ranged from 8.2 g/dL to 17.6 g/dL). Participants with anemia were older, more often males, and had a lower educational level and more chronic diseases than those with a normal hemoglobin level (Table 1). The levels of p-tau217, NfL, and GFAP were higher in participants with anemia than in those without (Table 1 and eFigure 2 in Supplement 1). Five measurements of p-tau217 below the limit of detection were replaced with a value of 0.

**Table 1.** Baseline Characteristics of the Study Population Overall and by Presence of Anemia

Characteristic	Participants^a^			*P* value
	Overall (n = 2282)	No anemia (n = 2083)	Anemia (n = 199)	
Demographics				
Age, y	72.2 (60.8-81.1)	72.1 (60.7-78.4)	81.4 (78.0-90.2)	<.001
Sex				
Female	1406 (61.6)	1301 (62.5)	105 (52.8)	.007
Male	876 (38.4)	782 (37.5)	94 (47.2)	
University education	826 (36.2)	776 (37.3)	50 (25.1)	<.001
*APOE* (≥1 ε4 allele)	652 (28.6)	593 (28.5)	59 (29.6)	.54
Chronic diseases				
No.	2.0 (1.0-4.0)	2.0 (1.0-3.0)	4.0 (3.0-6.0)	<.001
Chronic kidney disease	741 (32.5)	620 (29.8)	121 (60.8)	<.001
Ischemic heart disease	307 (13.5)	262 (12.6)	45 (22.6)	<.001
Heart failure	178 (7.8)	137 (6.6)	41 (20.6)	<.001
Atrial fibrillation	190 (8.3)	151 (7.2)	39 (19.6)	<.001
Cerebrovascular disease	132 (5.8)	107 (5.1)	25 (12.6)	<.001
Cancer	192 (8.4)	161 (7.7)	31 (15.6)	<.001
Underweight	54 (2.4)	41 (2.0)	13 (7.1)	<.001
AD blood biomarkers, pg/mL				
p-tau217	0.10 (0.06-0.18)	0.10 (0.05-0.16)	0.20 (0.11-0.34)	<.001
NfL	18.0 (12.5-28.3)	17.0 (12.3-26.0)	36.6 (23.2-53.7)	<.001
GFAP	121.1 (80.0-188.5)	117.4 (77.7-178.0)	187.8 (128.0-291.3)	<.001
Incident dementia cases	362 (16.9)	309 (15.9)	53 (27.0)	<.001

Abbreviations: AD, Alzheimer disease; GFAP, glial fibrillary acidic protein; NfL, neurofilament light chain; p-tau217, phosphorylated tau 217.

^a^ Data are reported as median (IQR) for continuous variables and as number (percentage) of participants for categorical variables. A total of 68 participants were missing data for *APOE*, 69 for underweight, and 1 for educational level.

### Hemoglobin Levels and Dementia Risk

During 16 years of follow-up (19 988 person-years; mean [SD] of 9.3 [4.3] years per person), 142 participants (6.2%) dropped out of the study and 362 (15.9%) developed dementia. On average, compared with those with follow-up data, participants who withdrew were younger (mean difference, –7.52 years; 95% CI, –9.27 to –5.76 years), were more educated (67 of 142 [47.2%] vs 759 of 2140 [35.4%] had university education), and had fewer chronic diseases (mean difference, –1.00 diseases; 95% CI, –1.39 to –0.60 diseases). The incidence rate of dementia was higher in participants with anemia (4.37 [95% CI, 3.34-5.72] per 100 person-years) than in those with normal hemoglobin (1.65 [95% CI, 1.47-1.84] per 100 person-years). In the basic model, anemia was associated with a higher hazard of dementia (hazard ratio [HR], 1.72; 95% CI, 1.27-2.32), which was slightly attenuated after adjustment for chronic diseases, underweight, IL-6 level, and iron and vitamin supplementation (HR, 1.66; 95% CI, 1.21-2.28).

 Anemia was associated with a higher dementia hazard in males than females (adjusted HR [AHR], 2.40 [95% CI, 1.39-4.11] vs 1.55 [95% CI, 1.03-2.33]) in fully adjusted models, though the interaction was not statistically significant. Anemia was associated with dementia among participants not carrying *APOE* ε4 (AHR, 1.60; 95% CI, 1.04-2.45) but not among carriers (AHR, 1.29; 95% CI, 0.76-2.21), with no significant interaction. The association between anemia and dementia remained after excluding participants with baseline MCI (n = 403 [17.7%]) (AHR, 1.89; 95% CI, 1.33-2.69) or those who developed dementia within the first 6 years (n = 177 [7.8%]) (AHR, 1.73; 95% CI, 1.07-2.81).

**Figure 1.**
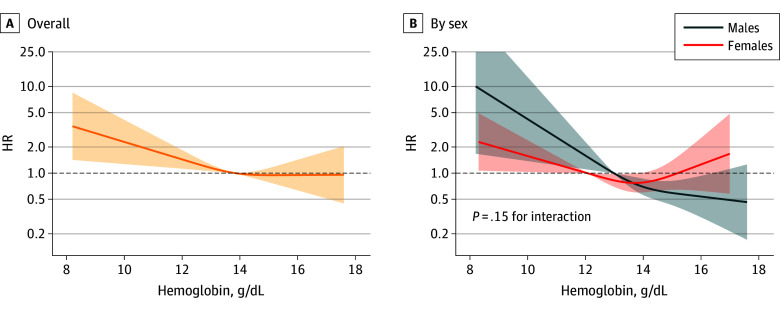
Line Graphs Showing Incident Dementia Risk by Hemoglobin Level at Baseline in the Overall Population and by Sex Lines represent adjusted hazard ratios (AHRs); shading indicates 95% CIs. Models adjusted for age cohort, sex, educational level, chronic kidney disease, heart disease, cerebrovascular disease, cancer, underweight, vitamin and iron supplements, and interleukin 6 level. Mean hemoglobin reference values were 13.8 g/dL for the overall population, 13.0 g/dL for males, and 12.0 g/dL for females (to convert to g/L, multiply by 10.0).

A nonlinear association was observed between baseline hemoglobin levels and incident dementia (Figure 1), with lower hemoglobin levels (up to about 14 g/dL) associated with a higher dementia risk, above which the association plateaued. In sex-stratified analysis, the association was significant in males but not females. No significant sex × hemoglobin or *APOE* × hemoglobin interaction was detected (Figure 1 and eFigure 3 in Supplement 1).

### Hemoglobin Levels and Blood Biomarkers of AD

**Figure 2.**
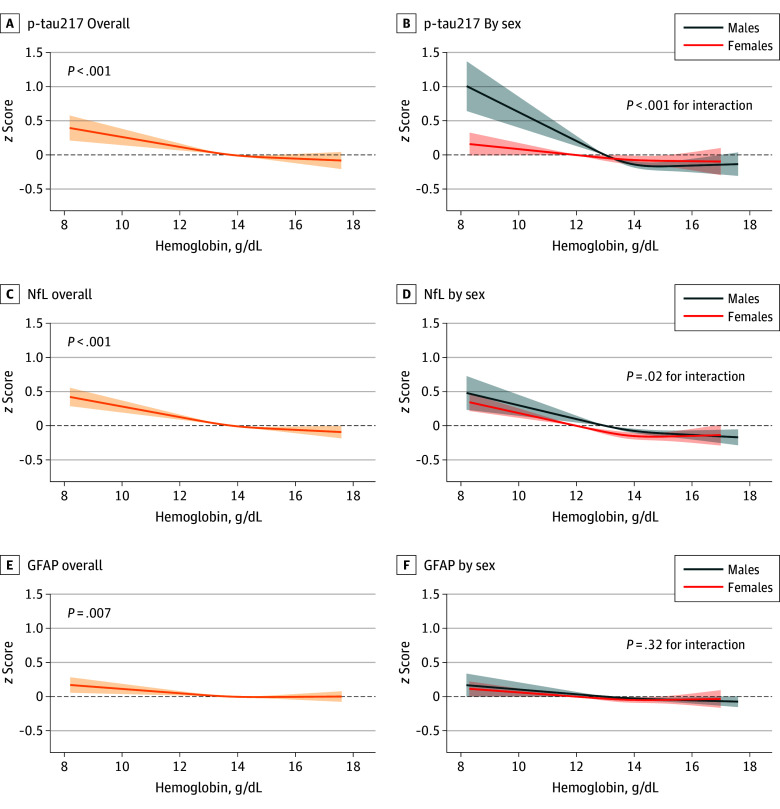
Line Graphs Showing Associations Between Hemoglobin Levels and Blood Biomarkers of Alzheimer Disease at Baseline Models adjusted for age cohort, sex, educational level, chronic kidney disease, heart disease, cerebrovascular disease, cancer, underweight, vitamin and iron supplements, and interleukin 6. Mean hemoglobin reference values were 13.8 g/dL overall, 13.0 g/dL for males, and 12.0 g/dL for females (to convert to g/L, multiply by 10.0). GFAP indicates glial fibrillary acidic protein; NfL, neurofilament light chain; p-tau217, phosphorylated tau 217.

We observed an inverse cross-sectional association between hemoglobin and all 3 AD blood biomarkers at baseline; individuals with lower hemoglobin levels displayed higher levels of p-tau217, NfL, and GFAP, following a nonlinear association (Figure 2). In fully adjusted models, the greatest elevation in the presence of anemia was shown by NfL (β, 0.25; 95% CI, 0.19-0.31), followed by p-tau217 (β, 0.22; 95% CI, 0.15-0.30), whereas GFAP was only slightly elevated (β, 0.08; 95% CI, 0.03-0.12).

Sex-stratified analyses (Figure 2) showed more pronounced elevations in males than females for p-tau217 (*P* < .001 for the hemoglobin × sex interaction) and, to a lesser extent, NfL (*P* = .02 for interaction). No interaction with *APOE* ε4 carrier status was observed (eFigure 4 in Supplement 1). Results remained consistent after excluding participants with baseline MCI or those who developed dementia within the first 6 years (eTables 2 and 3 in Supplement 1).

### Hemoglobin, Blood Biomarkers of Alzheimer Disease, and Dementia Risk

**Figure 3.**
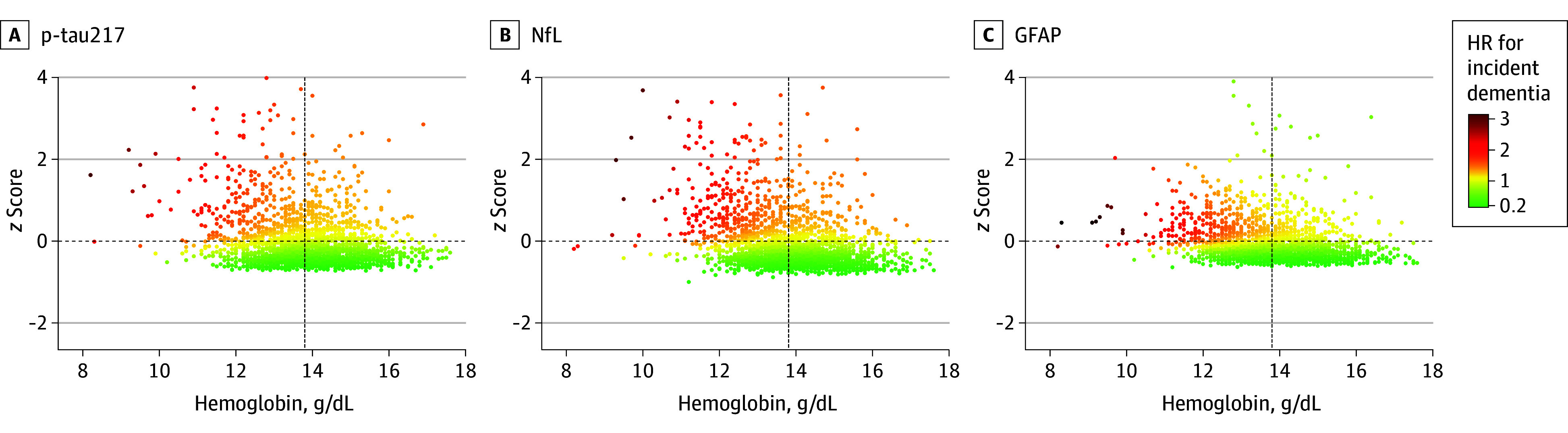
Scatter Plots of Adjusted Hazard Ratios (AHRs) for Incident Dementia by Continuous Levels of Hemoglobin and of Blood Biomarkers of Alzheimer Disease (AD) at Baseline Vertical dashed lines indicate mean hemoglobin level (13.8 g/dL; to convert to g/L, multiply by 10.0) and horizontal dashed lines, mean *z* score of the biomarker level (0). Models adjusted for age cohort, sex, educational level, chronic kidney disease, heart disease, cerebrovascular disease, cancer, underweight, vitamin and iron supplements, and interleukin 6 level. GFAP indicates glial fibrillary acidic protein; NfL, neurofilament light chain; p-tau217, phosphorylated tau 217.

In addition, we explored the joint association of hemoglobin and AD blood biomarker levels with dementia risk. As shown in Figure 3, participants with normal hemoglobin and low AD biomarker levels had the lowest dementia risk, which increased as hemoglobin decreased and AD biomarker levels increased (ie, upper left quadrant of the plots).

We observed an additive interaction between anemia and elevated NfL levels in association with incident dementia (Table 2). Using participants without anemia and with low NfL as the reference group, the AHR for dementia was 1.09 (95% CI, 0.43-2.75) among those with anemia only, 2.16 (95% CI, 1.60-2.91) among those with high NfL only, and 3.64 (95% CI, 2.39-5.56) among participants with both anemia and high NfL. Elevated dementia risk was also observed for anemia combined with high p-tau217 or GFAP levels; however, no additive interaction was detected for these biomarkers.

**Table 2.** AHRs of Dementia Associated With Presence or Absence of Anemia and With High vs Low Levels of Blood Biomarkers of Alzheimer Disease at Baseline

Joint exposures	AHR (95% CI)^a^		
	Overall	Females	Males
**p-tau217 and Anemia**			
Low p-tau217 + no anemia	1 [Reference]	1 [Reference]	1 [Reference]
Low p-tau217 + anemia	1.89 (1.05 to 3.39)	1.47 (0.63-3.41)	3.01 (1.27-7.09)
High p-tau217 + no anemia	2.11 (1.63 to 2.73)	2.15 (1.57-2.93)	1.96 (1.25-3.14)
High p-tau217 + anemia	3.01 (1.99 to 4.55)	2.91 (1.75-4.84)	3.94 (1.90-8.17)
Attributable proportion (95% CI)^b^	0.01 (−0.51 to 0.52)	NA	NA
**NfL and anemia**			
Low NfL + no anemia	1 [Reference]	1 [Reference]	1 [Reference]
Low NfL + anemia	1.09 (0.43 to 2.75)	0.36 (0.05-2.77)	2.53 (0.87-7.35)
High NfL + no anemia	2.16 (1.60 to 2.91)	2.15 (1.49-3.12)	2.34 (1.41-3.89)
High NfL + anemia	3.64 (2.39 to 5.56)	3.42 (2.03-5.77)	5.48 (2.63-11.42)
Attributable proportion (95% CI)^b^	0.38 (0.04 to 0.73)	NA	NA
**GFAP and anemia**			
Low GFAP + no anemia	1 [Reference]	1 [Reference]	1 [Reference]
Low GFAP + anemia	1.70 (0.90 to 3.24)	2.13 (0.88-5.15)	2.07 (0.79-5.42)
High GFAP + no anemia	2.10 (1.60 to 2.77)	1.89 (1.34-2.67)	2.73 (1.71-4.36)
High GFAP + anemia	3.34 (2.20 to 5.08)	2.56 (1.50-4.36)	7.16 (3.44-14.88)
Attributable proportion (95% CI)^b^	0.16 (−0.27 to 0.59)	NA	NA

Abbreviations: AHR, adjusted hazard ratio; GFAP, glial fibrillary acidic protein; NA, not applicable; NfL, neurofilament light chain; p-tau217, phosphorylated tau 217.

^a^ HRs were derived from Cox proportional hazards regression models, adjusted for age cohort, sex, educational level, chronic kidney disease, heart disease, cerebrovascular disease, cancer, underweight, vitamin and iron supplements, and interleukin 6 level. Blood biomarkers of Alzheimer disease were dichotomized using the following cutoffs: 0.13 pg/mL for p-tau217, 20.17 pg/mL for NfL, and 142.52 pg/mL for GFAP.

^b^ Attributable proportion refers to the additive interaction between anemia and the individual biomarker.

The AHRs for the joint associations were higher in males than in females (Table 2). Effect modification by *APOE* ε4 status was also observed (eTable 4 in Supplement 1). For instance, anemia combined with low p-tau217 was associated with a higher dementia risk among participants not carrying *APOE* ε4 (AHR, 2.64; 95% CI, 1.31-5.30) but not among carriers (AHR, 0.44; 95% CI, 0.10-1.88) (*P* = .06 for interaction). A statistically significant interaction with APOE-ε4 status was observed for NfL (*P* = .047 for interaction) but not for GFAP (*P* = .10 for interaction). Results remained consistent after excluding participants with baseline MCI or those who developed dementia within the first 6 years (eTables 5 and 6 in Supplement 1).

## Discussion

In this large population-based study, lower hemoglobin levels were associated with progressively higher dementia risk, following a nonlinear dose-response association. Individuals with anemia had a 66% higher hazard of dementia compared with those with a normal hemoglobin level. Low hemoglobin was also associated with elevated blood concentrations of AD biomarkers, particularly NfL and p-tau217, while increases in GFAP were less pronounced. Co-occurrence of low hemoglobin and elevated AD blood biomarkers was associated with further amplified dementia risk, suggesting a potential interplay between anemia and neuropathology. Anemia was associated with higher dementia risk and with higher levels of AD blood biomarkers in males than in females. Overall, our findings expand previous knowledge of the anemia-dementia association by suggesting an interplay between anemia and neuropathology—as measured by blood biomarkers—in dementia development.

Previous population-based studies have reported associations between anemia and dementia risk.^3,4,20,21,22,23,24^ Our findings align with a meta-analysis of 5 prospective population-based studies estimating that individuals with anemia had a higher risk of dementia than those without (risk ratio, 1.46; 95% CI, 1.22-1.76).^25^ Shared risk factors such as malnutrition, iron and vitamin deficiency,^26,27,28^ systemic inflammation, and chronic diseases (eg, chronic kidney disease)^29^ have been proposed to explain this association. However, in our study, adjusting for these potential confounders did not substantially attenuate the association, suggesting that these factors alone are unlikely to fully explain the increased risk of dementia—and the elevated p-tau217, NfL, and GFAP levels—observed in participants with low hemoglobin.

In a previous study by our group, anemia emerged as 1 of the chronic conditions most significantly associated with elevated levels of AD blood biomarkers.^12^ Expanding that evidence, in this study we also included p-tau217, currently regarded as the most specific blood biomarker for AD,^30^ and we observed that all biomarker levels tended to be higher as hemoglobin levels declined, following a nonlinear dose-response association. These findings suggest a more nuanced relation between hemoglobin levels, AD blood biomarkers, and dementia beyond the definition of anemia itself. Among the biomarkers, NfL and p-tau217 showed the most pronounced variations, in line with findings from the MEMENTO cohort,^31^ where hemoglobin was significantly associated with variability in blood p-tau181 and NfL among dementia-free individuals. While the association with NfL is somewhat expected, as anemia has previously been associated with neuronal loss and brain atrophy,^7,20^ the link between hemoglobin and AD pathology remains less clear. Indeed, although several studies reported an elevated risk of clinically diagnosed AD dementia in individuals with low hemoglobin,^3,21,23^ direct evidence linking anemia with biomarkers of AD pathology is limited and conflicting.^8,32^ A study by Yang et al^8^ reported an association of anemia with altered CSF levels of Aβ42 but not p-tau, whereas Kim et al^32^ did not find associations between hemoglobin and Aβ or tau deposition measured through PET imaging.

Beyond the associations of low hemoglobin with elevated dementia risk and with elevated AD blood biomarker levels, we also observed a joint association of anemia and AD blood biomarkers—particularly NfL—with dementia risk, with the highest risk observed among individuals with both low hemoglobin and elevated biomarkers. These findings lend themselves to several possible interpretations. One is that anemia may reduce brain resilience, thereby lowering the threshold at which neuropathology manifests clinically as dementia.^33,34^ Anemia could increase neuronal vulnerability through chronic cerebral hypoxia, which can trigger a cascade of pathologic processes, including oxidative stress, neuroinflammation, and progressive neuronal damage and loss.^6,35^ Consequently, the same level of biomarker-detected pathology may confer a higher risk of dementia among individuals with anemia. Alternatively, neuropathologic alterations reflected by elevated biomarker levels may represent a key mechanism linking anemia to dementia development. Elevated NfL levels may reflect neurodegenerative processes occurring in older adults with anemia,^7,20^ which could act as mediators in the association between anemia and dementia. Future studies should further investigate this possibility and formally assess whether—and which—blood biomarkers mediate the relationship between anemia and dementia development.

Anemia was associated with higher dementia risk and higher levels of AD blood biomarkers in males than in females, suggesting sex-specific vulnerability to low hemoglobin levels. While females tend to have lower hemoglobin levels and higher anemia prevalence early in life,^36,37^ often due to reproductive factors, anemia in males is less common, occurs later, and is frequently driven by chronic diseases, inflammation, or nutritional deficiencies.^38^ Females’ generally lower baseline hemoglobin levels might confer greater tolerance to anemia, buffering its impact on brain health.^39^ This study’s findings should be interpreted cautiously, as sex interactions were not always significant and prior studies have not directly examined sex-specific associations between hemoglobin, AD biomarkers, and dementia risk.

### Strengths and Limitations

To our knowledge, this is the first study to explore the link between hemoglobin, blood biomarkers of AD, and dementia. Strengths include the large population-based sample of dementia-free older adults with up to 16 years of follow-up; standardized and comprehensive data collection encompassing clinical, biological, and cognitive measures; and availability of multiple blood biomarkers reflecting different neuropathologic processes.

Some limitations should be mentioned. First, hemoglobin levels ranged between 8.2 g/dL and 17.6 g/dL, and 90.5% of anemia cases were normocytic, limiting our possibility to explore more extreme hemoglobin levels and microcytic or macrocytic anemia. Second, AD biomarkers were measured in serum, which typically yields lower concentrations than plasma. However, previous studies showed strong correlations between serum and plasma biomarker levels and comparable diagnostic accuracy.^40,41^ Third, a substantial proportion of participants lacked biomarker and hemoglobin data; they were generally older and less educated and had a higher burden of comorbidities than those included. Since they were likely at higher risk of anemia and dementia, their exclusion may have led to an underestimation of the associations. Fourth, study participants were predominantly White, limiting generalizability to more diverse populations. Also, blood biomarkers were only available at baseline, preventing us from exploring the association between hemoglobin and AD blood biomarkers over time.

## Conclusions

In this cohort study of dementia-free older adults, individuals with anemia exhibited higher risk of incident dementia, alongside elevated concentrations of AD-related blood biomarkers. Dementia risk was particularly high when anemia co-occurred with high levels of blood biomarkers reflecting AD pathology, neurodegeneration, and glial activation. This suggests a biological interplay between anemia and neuropathology, in which low hemoglobin may not only contribute to neuropathology but also reduce the brain’s resilience to it. Taken together, our findings suggest anemia is a clinically relevant factor in the context of dementia risk stratification and is possibly a modifiable target in dementia prevention strategies.

## Notes

Supplement 1. **eFigure 1.** Flow Chart of Study Participants **eFigure 2.** Levels of AD Blood Biomarkers by Presence of Anemia at Baseline **eFigure 3.** Association Between Hemoglobin Levels and Incident Dementia, Stratified by *APOE* ε4 Carrier Status **eFigure 4.** Cross-Sectional Association Between Hemoglobin Levels and Levels of AD Blood Biomarkers, Stratified by *APOE* ε4 Carrier Status **eTable 1.** Comparison of Baseline Characteristics Between Participants Included in the Analysis (n= 2282) and Those Excluded Due to Missing Data (n= 841) **eTable 2.** Cross-Sectional Association Between Anemia and Levels of AD Blood Biomarkers, Excluding Participants With Mild Cognitive Impairment (MCI) at Baseline (n = 403) **eTable 3.** Cross-Sectional Association Between Anemia and Levels of AD Blood Biomarkers, Excluding Participants Who Developed Dementia Within the First 6 Years of Follow-Up (N = 177) **eTable 4.** Hazard Ratios (HRs) of Dementia in Relation to the Presence or Absence of Anemia and to the Levels (High vs Low) of Blood Biomarkers of Alzheimer Disease at Baseline, Stratified by *APOE* ε4 Carrier Status **eTable 5.** Association of Anemia and AD Blood Biomarkers and Incident Dementia, Excluding Participants With Mild Cognitive Impairment (MCI) at Baseline (n = 403) **eTable 6.** Association of Anemia and AD Blood Biomarkers and Incident Dementia, Excluding Participants Who Developed Dementia Within the First 6 Years of Follow-Up (N = 177)

Supplement 2.
Data Sharing Statement
