# Supplementary material for: Anemia and Blood Biomarkers of Alzheimer Disease in Dementia Development
Source: JAMA Netw Open. 2026 Apr 17;9(4):e264029. doi: 10.1001/jamanetworkopen.2026.4029 (PMC13090852; doi:10.1001/jamanetworkopen.2026.4029)
Supplement: Supplement 1. — eFigure 1. Flow Chart of Study Participants eFigure 2. Levels of AD Blood Biomarkers by Presence of Anemia at Baseline eFigure 3. Association Between Hemoglobin Levels and Incident Dementia, Stratified by APOE ε4 Carrier Status eFigure 4. Cross-Sectional Association Between Hemoglobin Levels and Levels of AD Blood Biomarkers, Stratified by APOE ε4 Carrier Status eTable 1. Comparison of Baseline Characteristics Between Participants Included in the Analysis (n= 2282) and Those Excluded Due to Missing Data (n= 841) eTable 2. Cross-Sectional Association Between Anemia and Levels of AD Blood Biomarkers, Excluding Participants With Mild Cognitive Impairment (MCI) at Baseline (n = 403) eTable 3. Cross-Sectional Association Between Anemia and Levels of AD Blood Biomarkers, Excluding Participants Who Developed Dementia Within the First 6 Years of Follow-Up (N = 177) eTable 4. Hazard Ratios (HRs) of Dementia in Relation to the Presence or Absence of Anemia and to the Levels (High vs Low) of Blood Biomarkers of Alzheimer Disease at Baseline, Stratified by APOE ε4 Carrier Status eTable 5. Association of Anemia and AD Blood Biomarkers and Incident Dementia, Excluding Participants With Mild Cognitive Impairment (MCI) at Baseline (n = 403) eTable 6. Association of Anemia and AD Blood Biomarkers and Incident Dementia, Excluding Participants Who Developed Dementia Within the First 6 Years of Follow-Up (N = 177) [file jamanetwopen-e264029-s001.pdf]

Supplementary Online Content

Valletta M, Vetrano DL, Qiu C, et al. Anemia and blood biomarkers of Alzheimer disease in dementia development. *JAMA Netw Open*. 2026;9(3):e264029. doi:10.1001/jamanetworkopen.2026.4029

eFigure 1. Flow Chart of Study Participants

eFigure 2. Levels of AD Blood Biomarkers by Presence of Anemia at Baseline

eFigure 3. Association Between Hemoglobin Levels and Incident Dementia, Stratified by *APOE* ε4 Carrier Status

eFigure 4. Cross-Sectional Association Between Hemoglobin Levels and Levels of AD Blood Biomarkers, Stratified by *APOE* ε4 Carrier Status

eTable 1. Comparison of Baseline Characteristics Between Participants Included in the Analysis (n= 2282) and Those Excluded Due to Missing Data (n= 841)

eTable 2. Cross-Sectional Association Between Anemia and Levels of AD Blood Biomarkers, Excluding Participants With Mild Cognitive Impairment (MCI) at Baseline (n = 403)

eTable 3. Cross-Sectional Association Between Anemia and Levels of AD Blood Biomarkers, Excluding Participants Who Developed Dementia Within the First 6 Years of Follow-Up (N = 177)

eTable 4. Hazard Ratios (HRs) of Dementia in Relation to the Presence or Absence of Anemia and to the Levels (High vs Low) of Blood Biomarkers of Alzheimer Disease at Baseline, Stratified by *APOE* ε4 Carrier Status

eTable 5. Association of Anemia and AD Blood Biomarkers and Incident Dementia, Excluding Participants With Mild Cognitive Impairment (MCI) at Baseline (n = 403)

eTable 6. Association of Anemia and AD Blood Biomarkers and Incident Dementia, Excluding Participants Who Developed Dementia Within the First 6 Years of Follow-Up (N = 177)

This supplementary material has been provided by the authors to give readers additional information about their work.

© 2026 Valletta M et al. *JAMA Network Open*.

eFigure 1. Flow chart of study participants.

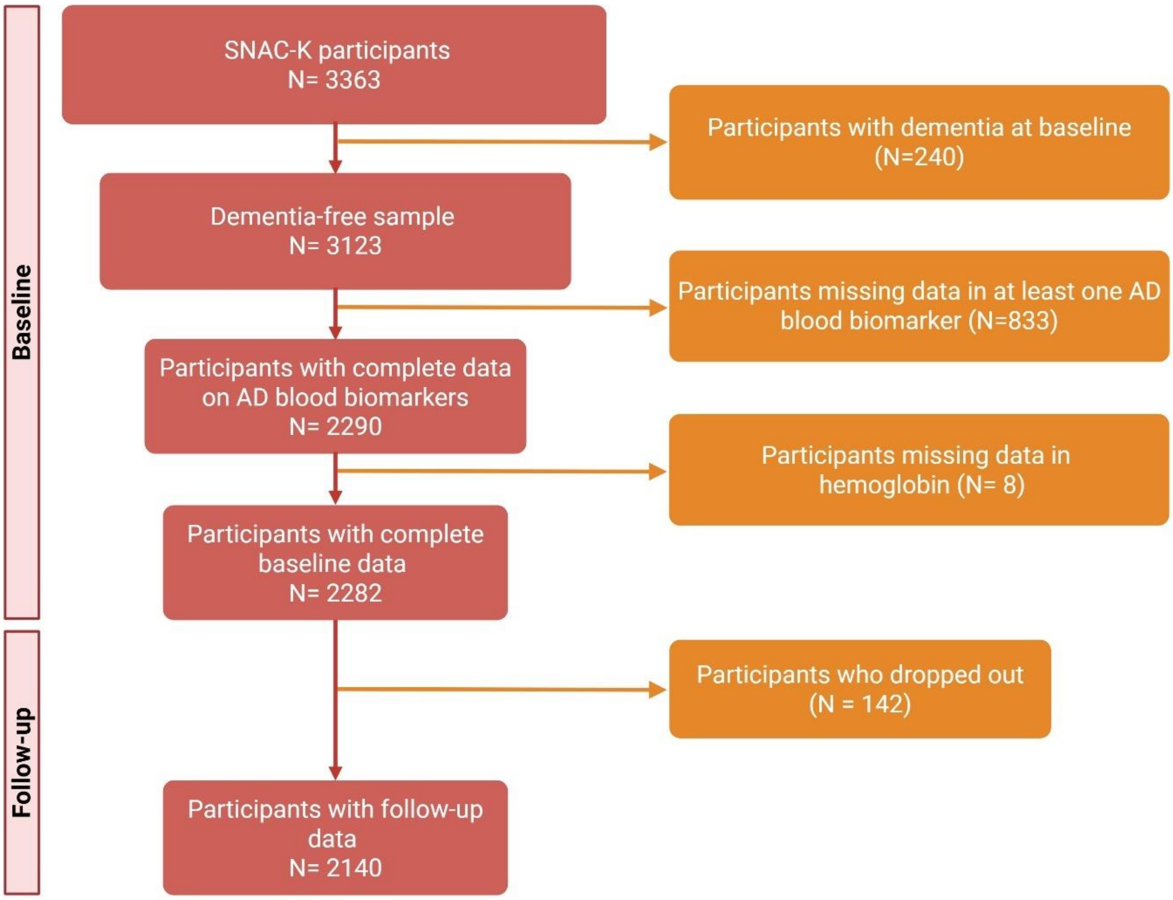

© 2026 Valletta M et al. *JAMA Network Open*.

eFigure 2. Levels of AD blood biomarkers by presence of anemia at baseline

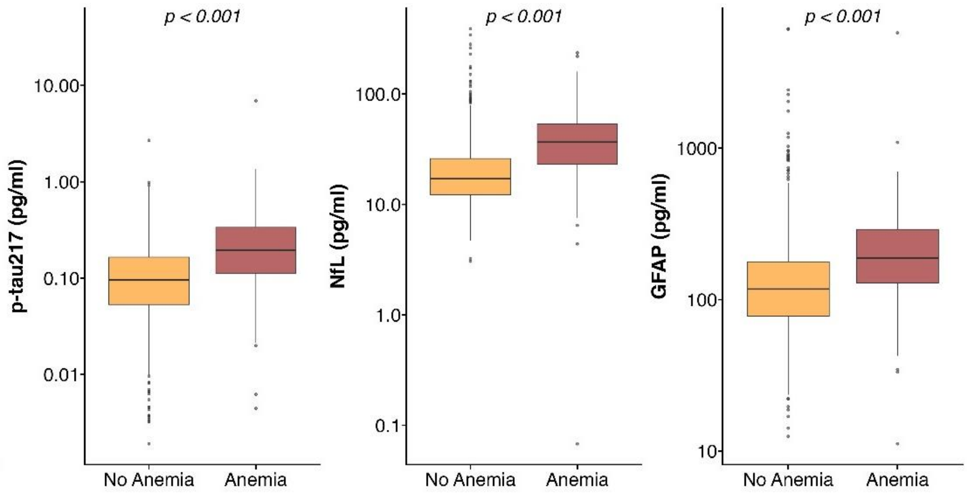

Box plots show the median (central line) and interquartile range (box) as well as the 2.5th and 97.5th percentiles (whiskers). P values are derived from Kruskal-Wallis test. Abbreviations: p-tau217: phosphorylated-tau217; NfL: neurofilament light chain; GFAP: glial fibrillary acidic protein. Some outliers were not represented for graphical purposes (n = 2 for NfL and 7 for GFAP).

© 2026 Valletta M et al. *JAMA Network Open*.

eFigure 3. Association between hemoglobin levels and incident dementia, stratified by *APOE* ε4 carrier status.

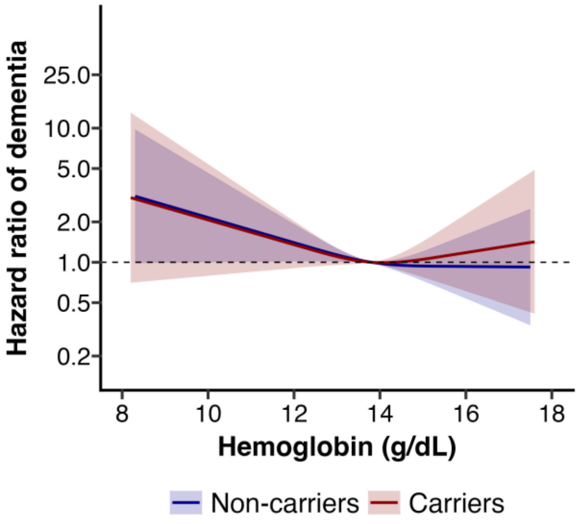

Cox regression models adjusted for age cohort, sex, education, chronic kidney disease, heart disease, cerebrovascular disease, cancer, underweight, vitamin and iron supplements and IL-6.

© 2026 Valletta M et al. *JAMA Network Open*.

eFigure 4. Cross-sectional association between hemoglobin levels and levels of AD blood biomarkers, stratified by *APOE* ε4 carrier status.

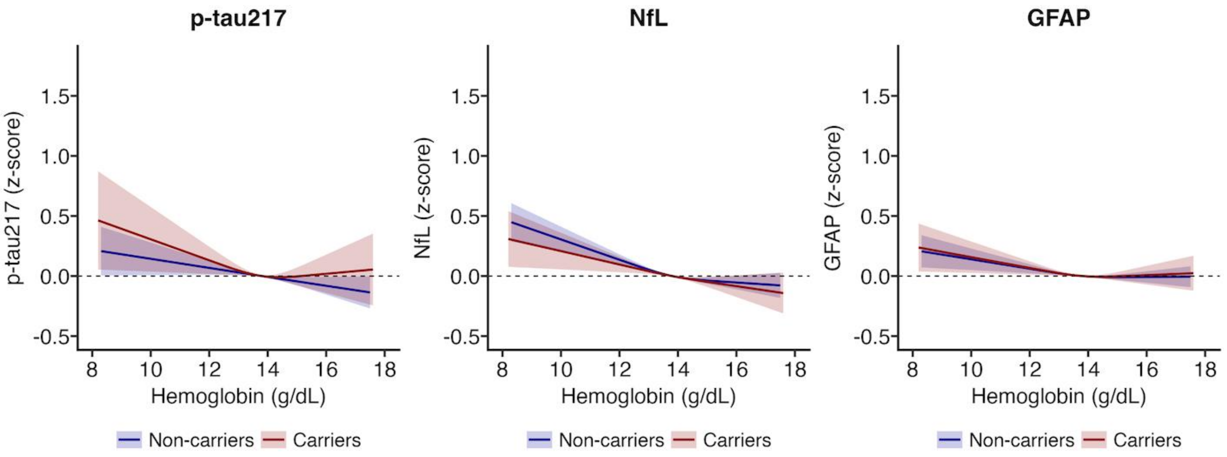

Quantile regression models on the median adjusted for age cohort, sex, education, chronic kidney disease, heart disease, cerebrovascular disease, cancer, underweight, vitamin and iron supplements and IL-6.

© 2026 Valletta M et al. *JAMA Network Open*.

eTable 1. Comparison of baseline characteristics between participants included in the analysis (n= 2282) and those excluded due to missing data (n= 841).

	Included N= 2282	Excluded N=841	p-value
Age	72.2 (60.8-81.1)	78.2 (66.5-84.5)	<0.001
Sex (F)	1,406 (61.6%)	573 (68.1%)	<0.001
Education (university or higher)	826 (36.2%)	239 (28.4%)	<0.001
APOE (at least one ε4 allele)	652 (29.4%)	167 (26.9%)	0.22
Number of chronic diseases	3.0 (2.0-5.0)	4.0 (2.0-6.0)	<0.001
Chronic kidney disease	741 (32.5%)	297 (35.3%)	0.13
Heart diseases	498 (21.8%)	230 (27.3%)	0.001
Cerebrovascular diseases	132 (5.8%)	82 (9.8%)	<0.001
Cancer	192 (8.4%)	87 (10.3%)	0.093

Data are reported as median (Q1-Q3) for continuous variables and as n (%) for categorical variables.

© 2026 Valletta M et al. *JAMA Network Open*.

eTable 2. Cross-sectional association between anemia and levels of AD blood biomarkers, excluding participants with mild cognitive impairment (MCI) at baseline (n = 403).

		(95% CI)
**P-tau217**		
-	No Anemia	Ref.
-	Anemia	0.21 (0.12, 0.29)
**NfL**		
-	No Anemia	Ref.
-	Anemia	0.26 (0.20, 0.32)
**GFAP**		
-	No Anemia	Ref.
-	Anemia	0.10 (0.05, 0.15)

Quantile regression models on the median adjusted for age cohort, sex, education, chronic kidney disease, heart disease, cerebrovascular disease, cancer, underweight, vitamin and iron supplements and IL-6.

© 2026 Valletta M et al. *JAMA Network Open*.

eTable 3. Cross-sectional association between anemia and levels of AD blood biomarkers, excluding participants who developed dementia within the first 6 years of follow-up (N = 177).

		ß (95% CI)
**P-tau217**		
-	No Anemia	Ref.
-	Anemia	0.15 (0.07, 0.23)
**NfL**		
-	No Anemia	Ref.
-	Anemia	0.20 (0.14, 0.26)
**GFAP**		
-	No Anemia	Ref.
-	Anemia	0.06 (0.01, 0.11)

Quantile regression models on the median adjusted for age cohort, sex, education, chronic kidney disease, heart disease, cerebrovascular disease, cancer, underweight, vitamin and iron supplements and IL-6.

© 2026 Valletta M et al. *JAMA Network Open*.

eTable 4. Hazard ratios (HRs) of dementia in relation to the presence or absence of anemia and to the levels (high vs low) of blood biomarkers of Alzheimer disease at baseline, stratified by *APOE* ε4 carrier status.

	APOE-ε4	
**Joint exposures**	Non carriers<br>HR (95% CI)	Carriers<br>HR (95% CI)
**P-tau217 and anemia**		
Low p-tau217 + no anemia	1 (Ref)	1 (Ref)
Low p-tau217 + anemia	2.64 (1.31, 5.30)	0.44 (0.10, 1.88)
High p-tau217 + no anemia	1.77 (1.27, 2.48)	1.83 (1.17, 2.87)
High p-tau217 + anemia	2.00 (1.13, 3.56)	4.11 (2.04, 8.28)
p interaction *APOE**joint exposure: 0.06		
**NfL and anemia**		
Low NfL + no anemia	1 (Ref)	1 (Ref)
Low NfL + anemia	1.71 (0.49, 5.97)	0.26 (0.04, 1.93)
High NfL + no anemia	2.51 (1.66, 3.82)	1.57 (0.99, 2.49)
High NfL + anemia	3.72 (2.08, 6.64)	2.96 (1.48, 5.94)
p interaction *APOE**joint exposure: 0.047		
**GFAP and anemia**		
Low GFAP + no anemia	1 (Ref)	1 (Ref)
Low GFAP + anemia	2.09 (0.92, 4.77)	0.62 (0.15, 2.65)
High GFAP + no anemia	1.96 (1.36, 2.84)	1.70 (1.09, 2.64)
High GFAP + anemia	2.75 (1.56, 4.86)	2.62 (1.33, 5.14)
p interaction *APOE**joint exposure: 0.10		

Hazard ratios are derived from Cox regression models, adjusted for age cohort, sex, education, chronic kidney disease, heart disease, cerebrovascular disease, cancer, underweight, vitamin and iron supplements and IL-6. Blood biomarkers of Alzheimer's disease were dichotomized using the following cut-offs: 0.134 pg/mL for p-tau217, 20.171 pg/mL for NfL and 142.515 pg/mL for GFAP. Abbreviations: GFAP: glial fibrillary acidic protein; NfL: neurofilament light chain; p-tau217: phosphorylated-tau217.

© 2026 Valletta M et al. *JAMA Network Open*.

eTable 5. Association of anemia and AD blood biomarkers and incident dementia, excluding participants with mild cognitive impairment (MCI) at baseline (n = 403).

**Joint exposures**	**HR (95% CI)**
**P-tau217 and anemia**	
Low p-tau217 + no anemia	Ref
Low p-tau217 + anemia	2.18 (1.14, 4.16)
High p-tau217 + no anemia	1.92 (1.42, 2.60)
High p-tau217 + anemia	3.12 (1.98, 4.92)
AP (95% CI)	0.00 (-0.58, 0.59)
**NfL and anemia**	
Low NfL + no anemia	Ref
Low NfL + anemia	1.27 (0.49, 3.34)
High NfL + no anemia	2.06 (1.45, 3.34)
High NfL + anemia	4.08 (2.52, 6.60)
AP (95% CI)	0.43 (0.06, 0.79)
**GFAP and anemia**	
Low GFAP + no anemia	Ref
Low GFAP + anemia	1.49 (0.69, 3.20)
High GFAP + no anemia	1.99 (1.44, 2.75)
High GFAP + anemia	3.94 (2.48, 6.26)
AP (95% CI)	0.37 (0.00, 0.74)

Cox regression models adjusted for age cohort, sex, education, chronic kidney disease, heart disease, cerebrovascular disease, cancer, underweight, vitamin and iron supplements and IL-6.

© 2026 Valletta M et al. *JAMA Network Open*.

eTable 6. Association of anemia and AD blood biomarkers and incident dementia, excluding participants who developed dementia within the first 6 years of follow-up (N = 177).

**Joint exposures**	**HR (95% CI)**
**P-tau217 and anemia**	
Low p-tau217 + no anemia	Ref
Low p-tau217 + anemia	1.78 (0.84, 3.78)
High p-tau217 + no anemia	1.81 (1.29, 2.54)
High p-tau217 + anemia	2.83 (1.47, 5.43)
AP (95% CI)	0.08 (-0.67, 0.83)
**NfL and anemia**	
Low NfL + no anemia	Ref
Low NfL + anemia	1.00 (0.35, 2.85)
High NfL + no anemia	2.05 (1.42, 2.96)
High NfL + anemia	4.38 (2.38, 8.07)
AP (95% CI)	0.53 (0.18, 0.89)
**GFAP and anemia**	
Low GFAP + no anemia	Ref
Low GFAP + anemia	1.48 (0.62, 3.57)
High GFAP + no anemia	2.36 (1.66, 3.35)
High GFAP + anemia	4.49 (2.40, 8.38)
AP (95% CI)	0.37 (-0.10, 0.83)

Cox regression models adjusted for age cohort, sex, education, chronic kidney disease, heart disease, cerebrovascular disease, cancer, underweight, vitamin and iron supplements and IL-6.
