# Supplementary material for: Anemia and Blood Biomarkers of Alzheimer Disease in Dementia Development
Source: JAMA Netw Open. 2026 Apr 17;9(4):e264029. doi: 10.1001/jamanetworkopen.2026.4029 (PMC13090852; doi:10.1001/jamanetworkopen.2026.4029)
Supplement: Supplement 2. — Data Sharing Statement [file jamanetwopen-e264029-s002.pdf]

Data Sharing Statement

Valletta. Anemia and Blood Biomarkers of Alzheimer Disease in Dementia Development. *JAMA Netw Open*. Published March 30, 2026. doi:10.1001/jamanetworkopen.2026.4029

Data
Data available: Yes
Data types: Deidentified participant data
How to access data: SNAC-K data can be requested by qualified researchers at https://www.snac-k.se/.
When available: With publication

Supporting Documents
Document types: None

Additional Information
Who can access the data: Data will be made available to researchers whose proposed use of the data has been approved from the SNAC-K coordination group.
Types of analyses: Data will be made available only for the purpose specified in the application.
Mechanisms of data availability: Data will be available after the approval from the SNAC-K coordination group.
